# Predictive biomarkers for immunotherapy response in extensive-stage SCLC

**DOI:** 10.1007/s00432-023-05544-x

**Published:** 2024-01-20

**Authors:** Lin Zhu, Jing Qin

**Affiliations:** 1 Department of Thoracic Medical Oncology, Zhejiang Cancer Hospital, Hangzhou Institute of Medicine (HIM), Chinese Academy of Sciences, Hangzhou, 310022 Zhejiang China; 2 Zhejiang Key Laboratory of Diagnosis and Treatment Technology on Thoracic Oncology (Lung and Esophagus), Zhejiang Cancer Hospital, Hangzhou, 310022 People’s Republic of China

**Keywords:** SCLC, Immunotherapy, Biomarkers, Molecular subtypes, ctDNA

## Abstract

**Background:**

Small cell lung cancer (SCLC) accounts for about 13–15% of all lung cancers, and about 70% of SCLC patients have developed extensive-stage small cell lung cancer (ES-SCLC) at the time of diagnosis because of its highgrade malignancy, easy invasion, and metastasis. In recent years, immunotherapy combined with chemotherapy has become the standard first-line treatment for ES-SCLC. However, SCLC is a relatively immune-cold lung cancer subtype with a limited number of beneficiaries and a short benefit period. Therefore, the use of biomarkers to identify populations with significant benefits from immunotherapy will help improve the efficacy and survival benefits of immunotherapy. However, predictive biomarkers suitable for clinical practice have not been established in the field of SCLC.

**Purpose:**

In order to find the predictive biomarkers of immunotherapy for ES-SCLC, we summarized the research progress of traditional biomarkers, such as programmed cell death ligand 1 (PD-L1) and tumor mutation burden (TMB), and summarizes the research of potential biomarkers associated with prognosis, such as molecular subtypes, special gene expression, expression of major histocompatibility complex (MHC) I and II classes, tumor immune microenvironment (TIME), and circulating tumor DNA (ctDNA) .We aim to provide new insights on biomarkers.

**Conclusion:**

The exploration of biomarkers for immunotherapy of SCLC is still very difficult, and it is clear that conventional predictive biomarkers are not suitable for SCLC. At present, the molecular subtypes defined from transcription factors may have some guiding significance, which still needs to be confirmed by prospective clinical studies. In addition, the ctDNA positivity rate of SCLC is higher than that of other tumor types, which can also solve the dilemma of the difficulty of obtaining specimens of SCLC tissues. And the dynamic change of ctDNA also has great potential to predict the curative effect of SCLC, which is worth further clinical exploration.

## Introduction

Among new cancer cases, lung cancer is the second most prevalent (11.4%) and the first most mortality (18%) worldwide, according to GLOBOCAN (Sung et al. 2021). According to the pathological types, lung cancer is divided into two subtypes: non-small cell lung cancer (NSCLC) and small cell lung cancer (SCLC). Among them, SCLC accounts for about 13–15% of all lung cancers. Due to its high degree of malignancy and easy of invasion and metastasis, about 70% of SCLC patients have developed extensive small-cell lung cancer (ES-SCLC) at the time of diagnosis. In previous studies, ES-SCLC was highly sensitive to platinum chemotherapy (carboplatin or cisplatin) with etoposide in standard first-line treatment, with response rates of 60%–65% (Horn et al. 2018). Despite the high response rate, the prognosis of ES-SCLC patients was poor, with a median overall survival (mOS) of 9–11 months, a 1-year overall survival (OS) rate of 35%, a 2-year OS rate not exceeding 5%, and a 5-year OS rate of only 3% (Rudin et al. 2021; Farago and Keane 2018; Byers and Rudin 2015).

SCLC has a high mutation rate, suggested that these tumors may be immunogenic and may respond to immune checkpoint inhibitors (ICIs). Therefore, the addition of immunotherapy to chemotherapy may enhance anti-tumor immunity and potentially improve outcomes (Horn et al. 2018). In recent years, ES-SCLC has benefited most from chemotherapy plus immunotherapy, with mOS reaching 12.3–15.4 months (Horn et al. 2018; Cheng et al. 2022; Paz-Ares et al. 2019a). Given the results of two clinical trials, IMpower133 and CASPIAN, the combination of atezolizumab/durvalumab with platinum-based chemotherapy is the standard first-line therapy for ES-SCLC. Based on the study of IMpower133, compared with standard chemotherapy, atezolizumab combination with standard chemotherapy significantly increased median progression-free survival (mPFS) (5.2 vs. 4.3 months, HR: 0.77, 95% CI 0.62–0.96; *p* = 0.02) and mOS (12.3 vs. 10.3 months, HR: 0.70, 95% CI 0.54–0.91; *p* = 0.007) (Horn et al. 2018) and improved the OS rate at 12-months (51.9% vs. 39.0%) and 18-months (34.0% vs. 21.0%) (Liu et al. 2021). The CASPIAN study suggested that, compared with standard chemotherapy, durvalumab plus platinum-etoposide significantly increased the mOS (12.9 vs. 10.5 months, HR: 0.71, 95% CI 0.60–0.86; *p* = 0.0003) and improved the OS rate at 12 months (52.8% vs. 39.3%) and 24 months (22.9% vs. 13.9%). Meanwhile, it also showed that the OS rate at 36 months significantly increased by three times (17.6% vs. 5.8%), further improving the survival rate of ES-SCLC (Paz-Ares 2021; Paz-Ares et al. 2022). In CASPIAN study, the mPFS could not be tested for significance between the durvalumab plus platinum-etoposide group and the platinum-etoposide group (5.1 vs. 5.4 months, HR: 0.78, 95% CI 0.65–0.94) (Paz-Ares et al. 2019). This result was different from that of the IMpower133 study, which may be related to the fact that the CASPIAN study was designed as an open-label study and the treatment cycles and modalities were different between the two groups. The durvalumab plus platinum-etoposide group received up to four cycles of platinum-etoposide plus durvalumab, whereas the platinum-etoposide group received up to six cycles of platinum-etoposide plus prophylactic cranial irradiation (investigator’s discretion) (Paz-Ares et al. 2019; Goldman et al. 2021). In the ASTRUM-005 study, serplulimab, a fully humanized IgG4 monoclonal antibody against the PD-1 receptor, combined with chemotherapy significantly improved overall survival compared with chemotherapy alone (15.4 vs. 10.9 months, HR: 0.63, 95% CI 0.49–0.82; *p* < 0.001). Moreover, the estimated 12-month (60.7% vs. 47.8%) and 24-month (43.1% vs. 7.9%) OS rates were significantly enhanced, which confirmed for the first time that PD-1 monoclonal antibody combined with chemotherapy also achieved positive results and refreshed the long-term survival benefit record of patients with ES-SCLC. (Cheng et al. 2022).

However, there are still some problems to overcome: in both IMpower133 and CASPIAN, the mPFS and mOS were not very satisfying. The survival curves for both OS and progression-free survival (PFS) were intertwined for the first 6 months, which roughly corresponded to the duration of chemotherapy in both groups, and then separated gradually over time as the immunotherapy worked successfully in limited patients. This not only reflected the slow effect of immunotherapy, but also suggested that immunotherapy did not work to prolong survival of some patients. (Horn et al. 2018; Paz-Ares et al. 2019a; Liu et al. 2021; Paz-Ares 2021). Even though ASTRUM-005 further prolonged the survival of the patients, both the PFS and OS curves also intertwined for the first 2 months and 4 months, respectively (Cheng et al. 2022). All these indicate that the benefit of immunotherapy is confined to a limited proportion of SCLC patients. Therefore, the population characteristics need to be subdivided and recognized. It is an inspiring research direction that biomarkers for predicting the efficacy of immunotherapy can be used to screen out patients who may really benefit from immunotherapy, especially when the benefits of programmed cell death-1/programmed cell death ligand 1 (PD-1/PD-L1) ICIs in ES-SCLC are limited in the whole population.

Therefore, in order to help screen ES-SCLC patients who are likely to respond positively to ICIs, this review summarizes the research progress of biomarkers.

## Biomarkers

### PD-L1

The expression level of PD-L1 assessed by immunohistochemistry (IHC) is regarded as one of the possible biomarkers for predicting the response of NSCLC to ICIs (Keppens et al. 2021). Several phase III clinical trials of first-line immunotherapy combined with chemotherapy for SCLC showed that PD-L1 expression could not accurately predict the survival benefit of immunotherapy. In the subsequent analysis of the IMpower133 study (Liu et al. 2021), 137 cases (34% of the intention-to-treat [ITT] population) underwent biopsy and PD-L1 expression (PD-L1 Ventana SP263) were analyzed. Due to the limited number of patients whose tumors had PD-L1 expression ≥ 5%, the researchers attempted to use PD-L1 expression of tumor cells (TCs)/immune cells (ICs) at 1% and 5% as cut-off values to analyze the effect of PD-L1 expression on survival. In patients with PD-L1 expression of TCs or ICs < 1% (*n* = 65) and ≥ 1% (*n* = 72), the mOS in the atezolizumab and placebo groups was 10.2 vs 8.3 months (HR: 0.51, 95% CI, 0.30–0.89; *p* = 0.015), 9.7 vs 10.6 months (HR: 0.87, 95% CI 0.51–1.49; *p* = 0.607). In patients with PD-L1 expression of TCs or ICs < 5% (*n* = 108) and ≥ 5% (*n* = 29), the mOS of the atezolizumab and placebo groups was 9.2 vs 8.9 months (HR: 0.77, 95% CI 0.51–1.17; *p* = 0.2278), 21.6 vs 9.2 months (HR: 0.60, 95% CI 0.25–1.46; *p* = 0.2527), respectively. Although patients with PD-L1 expression of ≥ 5% in TCs or ICs had longer mOS in the atezolizumab group, no significant difference was observed in the PD-L1 subgroup analysis. The results showed that the expression level of PD-L1 could not be used as a predictive biomarker of immunotherapy (Table 1). Similarly, in the CASPIAN study (Paz-Ares 2019; Paz-Ares, et al. 2023), among 227 patients (42% of the ITT population) with evaluable PD-L1 levels, 1% were located with ICs and TCs cut-off values, respectively, and the expression level of PD-L1 also failed to distinguish differences in OS benefits. In the KEYNOTE-604 trial (Rudin et al. 2020), the combined positive score (CPS) was used to evaluate the status of PD-L1 expression (22C3 antibody), which did not support PD-L1 as a valuable biomarker to predict efficacy outcomes (Table 2). To analyze the possible reasons, firstly, the lower positive rate of SCLC-caused PD-L1 expression level may affect its predictive value for immunotherapy in SCLC patients. Among the 137 patients in the IMpower133 study, the proportion of the population with PD-L1 expression level ≥ 1% on TCs was 5.8%, and the PD-L1 expression level ≥ 1% was 50.4% on ICs (Reck et al. 2019). The PD-L1 analysis in the CASPIAN study also showed similar characteristics. In this study, the proportions of PD-L1 expression on TCs and on ICs ≥ 1% were 5.1% and 22.4%, respectively (Paz-Ares, et al. 2023; Paz-Ares, et al. 2019b). Secondly, neither the IMpower133 study nor the CASPIAN study had mandatory biopsy to obtain tissue, and the analyses related to the prediction of PD-L1 expression on the efficacy of immunotherapy were post hoc and not prospective, so only one-third of the samples evaluated for PD-L1 expression. And it was unclear whether the distribution characteristics of baseline characteristics of the two groups were balanced. The ASTRUM-005 trial (Cheng 2022) used PD-L1 expression levels (negative: tumor proportion score (TPS) < 1%, positive: TPS ≥ 1%, or not evaluable/unavailable) as a stratification factor, which can help us understand the prediction of PD-L1 value. However, from the subgroup analysis of OS (Cheng et al. 2022), the proportion of group with the TPS ≥ 1% was 16.4%, and the HR of the serplulimab group versus the placebo group was 0.92 (*p* = 0.44, 95% CI 0.44–1.89), suggesting that the PD-L1 expression level also could not distinguish the difference in OS benefit (Table 2). For now, PD-L1 is not sufficient to be a reliable biomarker in ES-SCLC.

**Table 1:** The mOS for subgroups with different PD-L1 expression levels in IMpower133 (Liu et al. 2021)

Subgroup	*N*	mOS (months)		HR (95% CI), *p* value
		EP + A arm	EP + placebo arm	
ITT	403	12.3	10.3	0.76 (0.60–0.95), 0.0154
BEP	137	9.9	8.9	0.70 (0.48–1.02), N/A
PD-L1 expression 1%				
TCs/ICs < 1% PD-L1	65	10.2	8.3	0.51 (0.30–0.89), 0.0150
TCs/ICs ≥ 1% PD-L1	72	9.7	10.6	0.87 (0.51–1.49), 0.6070
PD-L1 expression 5%				
TCs/ICs < 5% PD-L1	108	9.2	8.9	0.77 (0.51–1.17), 0.2278
TCs/ICs ≥ 5% PD-L1	29	21.6	9.2	0.60 (0.25–1.46), 0.2527

*N, Number of assessable persons; *mOS*, median overall survival; *HR*, hazard ratio, *HR* is the hazard ratio of the atezolizumab plus carboplatin-etoposide group compared with the carboplatin-etoposide plus placebo group; *ITT*, intention-to-treat population; *BEP*, biomarker-evaluable population; *EP*, carboplatin-etoposide; *A*, atezolizumab; *PD-L1*, programmed death ligand 1; *TCs*, tumor cells; *ICs*, tumor-infiltrating immune cells; *N/A*, not available; PD-L1 expression was tested by PD-L1 (SP263) immunohistochemistry assay

**Table 2:** Summary in the experiment of different PD-L1 expression level and the group HRs of OS and PFS

Trials	ICIS	Antibodies	PD-L1 EXPRESSION	N1	MOS, months (HR, 95% CI)	N2	MPFS, months (HR, 95% CI)
CASPIAN (Paz-Ares, et al. 2023)	Durvalumab	SP263	TCs < 1% and ICs < 1%	87/114	11.8 vs.10.2 (0.63, 0.47–0.85)	101/114	N/A (0.75, 0.56–1.00)
			TCs or ICs ≥ 1%	30/38	14.6 vs.12.2 (0.61, 0.37–1.02)	33/38	N/A (0.55, 0.33–0.93)
KEYNOTE-604 (Rudin et al. 2020)	Pembrolizumab	22C3	CPS < 1%	146/175	N/A (0.80, 0.58–1.11)	159/174	N/A (0.73, 0.54–1.01)
			CPS ≥ 1%	134/185	N/A (0.84, 0.60–1.18)	154/184	N/A (0.68, 0.49–0.94)
ASTRUM-005 (Cheng et al. 2022)	Serplulimab	22C3	TPS < 1%	121/317	15.0 vs. 10.5 (0.58, 0.44–0.76)	N/A	N/A
			TPS ≥ 1%	22/62	NR vs. 12.9 (0.92, 0.44–1.89)	N/A	N/A
			Not available	3/10	NR vs. 14.2 (0.42, 0.10–1.72)	N/A	N/A

*ICIS immune checkpoint inhibitors, *Antibodies* PD-1/L1 immunohistochemical detection antibodies, *N1* Number of events/participants of immunotherapy group in OS analysis, *N2* Number of events/participants of immunotherapy group in PFS analysis, *MOS* Median overall survival, *HR* Hazard ratio, *HR* is the hazard ratio of the immunotherapy plus chemotherapy group compared with the chemotherapy plus placebo group, *PD-L1* programmed death ligand 1, *TCs* tumor cells, *ICs* tumor-infiltrating immune cells, *CPS* combined positive score, *TPS* tumor proportion score, *N/A* not available, *NR* not reached

### Tumor mutation burden (TMB)

TMB is a biomarker for the outcome of immunotherapy in various tumor types, including lung cancer (Hellmann et al. 2018a; Yarchoan et al. 2017), but the use of TMB to predict the efficacy of immunotherapy for SCLC is still controversial, and the results of different studies are inconsistent (Sholl et al. 2020). In the IMpower133 study (Liu et al. 2021), peripheral blood tumor mutational burden (bTMB) was an exploratory endpoint for the analysis of studies using established cutoff values (≥ 16 vs. < 16 and ≥ 10 vs. < 10 mutations/Mb). Of the 374 patients tested, 351 cases (173 in the atezolizumab arm vs. 178 in the placebo arm) had high-quality TMB data for analysis. However, in the established bTMB cut-off groups, there was no difference in the benefit of PFS and OS, indicating that bTMB could not predict the efficacy of immunotherapy for ES-SCLC patients (Table 3). Similarly, JW Goldman et al. (Goldman 2020) explored the association of TMB with efficacy in the ITT population in patients with long-term benefit based on an exploratory analysis of the CASPIAN trial. Tissue tumor mutational burden (tTMB) levels were assessed using the FoundationOne CDx assay. 805 ES-SCLC patients were randomly divided into three subgroups: D + EP, D + T + EP, and EP (D: Durvalumab, T: Tremelimumab, EP: platinum–etoposide) according to a ratio of 1:1:1. In the three subgroups, 35% of patients in the ITT population were evaluable for tTMB, indicating that tTMB did not predict the difference in efficacy (OS, PFS, or objective response rate (ORR)) between D ± T + EP and EP (Table 4). However, in the Checkmate032 study (Hellmann et al. 2018b), TMB was measured in 211 patients, including 133 in the nivolumab group and 78 in the nivolumab plus ipilimumab group. Mutations with lower than 143 mutations, 143–247 mutations, and greater than 247 mutations were defined as low, medium, and high mutations. The mOS of the low, medium, and high mutation groups in the nivolumab group were 3.1 vs. 3.9 vs. 5.4 months (95% CI 2.4–6.8, 2.4–9.9, 2.8–8.0), respectively, and the mPFS were 1.3 vs. 1.3 vs. 1.4 months (95% CI 1.2–1.4, 1.2–1.4, 1.3–2.7). The mOS of the nivolumab plus ipilimumab group was 3.4 vs. 3.6 vs. 22.0 months (95% CI 2.8–7.3, 1.8–7.7, 8.2–NR), and the mPFS were 1.5 vs. 1.3 vs. 7.8 months (95% CI 1.3–2.7, 1.2–2.1, 1.8–10.7) (Table 5). It showed that TMB levels can predict the efficacy of nivolumab + /– ipilimumab immunotherapy to some extent, and patients with high TMB tend to benefit more. However, this study was only able to demonstrate the potential of TMB as a biomarker in second-line immune monotherapy, and did not demonstrate its predictive effect in first-line immunotherapy combined chemotherapy. It is difficult to accurately assess the correlation between TMB and immunotherapy efficacy with the combined treatment mode as that chemotherapy may induce the increase of TMB values (Cao et al. 2020; Crisafulli et al. 2022). Thus, the addition of chemotherapy may dilute the effect of TMB on immunotherapy. For now, neither bTMB nor tTMB could effectively predict the efficacy of immunotherapy combined chemotherapy.

**Table 3:** Relationship between bTMB expression and survival in the atezolizumab group and placebo group in IMpower 133 (Liu et al. 2021)

bTMB (mutations/Mb)	N	mOS (months)		HR (95% CI)
		CP/ET + A arm	CP/ET + placebo arm	
< 10	134	11.8	9.4	0.73 (0.49–1.08)
≥ 10	212	14.9	11.2	0.73 (0.53–1.00)
< 16	266	12.5	10.0	0.79 (0.60–1.04)
≥ 16	80	17.1	11.9	0.58 (0.34–0.99)

*N Number of assessable persons, *CP/ET* carboplatin plus etoposide, *A* atezolizumab, *bTMB* blood tumor mutational burden, *HR* hazard ratio, *HR* is the hazard ratio of the immunotherapy plus chemotherapy group compared with the chemotherapy plus placebo group, *mOS* median overall survival

**Table 4:** HRs of OS in subgroups with different tTMB expression levels in CASPIAN (Goldman 2020)

tTMB (mutations/Mb)	N	EP + D arm VS EP arm
		OS HR (95% CI)
< 8	68	0.75 (0.45–1.26)
≥ 8	110	0.71 (0.47–1.09)
< 10	95	0.77 (0.50–1.20)
≥ 10	83	0.68 (0.42–1.14)
< 12	111	0.80 (0.53–1.20)
≥ 12	67	0.65 (0.37–1.15)
< 14	137	0.76 (0.53–1.10)
≥ 14	41	0.62 (0.30–1.32)

*N Number of assessable persons, *EP* carboplatin/etoposide, *D*, durvalumab, *tTMB*, tissue tumor mutation burden; *HR* hazard ratio, *HR* is the hazard ratio of the immunotherapy plus chemotherapy group compared with the chemotherapy plus placebo group; OS, overall survival

**Table 5:** Relationship between TMB expression and survival in the nivolumab and nivolumab plus ipilimumab group in Checkmate032 (Hellmann et al. 2018b)

TMB (mutations)	Nivolumab arm			Nivolumab + ipilimumab arm		
	*N*	mPFS (months), 95% CI	mOS (months), 95% CI	*N*	mPFS (months), 95% CI	mOS (months), 95% CI
< 143	42	1.3, 1.2–1.4	3.1, 4–6.8	27	1.5, 1.3–2.7	3.4, 2.8–7.3
143–247	44	1.3, 1.2–1.4	3.9, 2.4–9.9	25	1.3, 1.2–2.1	3.6, 1.8–7.7
> 247	47	1.4, 1.3–2.7	5.4, 2.8–8.0	26	7.8, 1.8–10.7	22.0, 8.2-NR

*N Number of assessable persons, *TMB* tumor mutation burden, *mOS* median overall survival, *mPFS* median progression-free survival, *NR* not reached

### Molecular subtypes and gene expression

SCLC has distinct transcriptional subtypes, and the different subtypes may respond differently to immunotherapy. Professor Rudin et al. (Rudin et al. 2019) first divided SCLC into four subtypes: SCLC-A, SCLC-N, SCLC-P, and SCLC-Y according to the relative expression of four key transcriptional regulators: achaete-scute homologue 1 (ASCL1), neurogenic differentiation factor 1 (NEUROD1), POU class 2 homeobox 3 (POU2F3), and yes-associated protein 1 (YAP1). However, Baine et al. (Baine et al. 2020) failed to identify YAP1 as a specific expression isoform using IHC analysis, which was scattered throughout the other three types of SCLC. Therefore, Gay et al. (Gay et al. 2021; Gay et al. 2019) defined SCLC with low expression of ASCL1, NEUROD1, and POU2F3 transcription factors and associated with inflammatory gene signatures as a new subtype of the inflamed SCLC subtype (SCLC-I).

Chen et al. (Chen, et al. 2021) also classified SCLC samples without significant survival difference into four subtypes based on gene expression data—cluster 1 to 4, where cluster 2 and 3 corresponded to SCLC-A and SCLC-N in the subtypes of Gay et al., respectively, and the subtype expressed as the protein of clara cell secretory protein (CCSP) was categorized as cluster 1; The subtype with the lower expression of ASCL1 and NEUROD1, but higher expression of POU2F3 and NOTCH2 was categorized as cluster 4, which was also named “immune subtype” because of its immune-related features. And by building random forest models, POU2F3 was the up-regulated gene with the highest expression in immune subtype. In addition, through the analysis of sample data from previous studies, it was further found that this immune subtype included the previously classified the SCLC-I subtype and the SCLC-P subtype. The study also collected a cohort containing 28 relapsed SCLC samples from patients receiving immunotherapy or immunotherapy combined with chemotherapy and immunohistochemical staining of POU2F3 in these specimens. They found that SCLC patients with high POU2F3 expression had a significantly increased ORR to immunotherapy (AUC = 0.813), and POU2F3 protein levels were positively correlated with patient prognosis (*p* = 0.022). And two SCLC patients with high POU2F3 levels had significant regression of lung lesions, indicating that there may be strong infiltration of ICs in POU2F3-high SCLC. These conclusions collectively support the hypothesis that patients with high POU2F3 were more sensitive to immunotherapy, and SCLC-I subtype and SCLC-P subtype had the potential to serve as a predictive biomarker for SCLC immunotherapy.

However, Gay et al. (Gay et al. 2021) applied their proposed molecular subtypes to the samples in the IMpower133 study, and found through analysis that although all subtypes in the immunotherapy group showed the improvement trend, SCLC-P subtype had the worst survival data in each group compared with the other three subtypes (Table 6). This discrepancy might be associated with the small sample size included in the study. Consequently, further explorations with larger samples are still needed. In addition, we found the greatest survival benefit for SCLC-I compared to all other subtypes in the immunotherapy group (18.2 vs. 10.4 months, HR: 0.57, 95% CI 0.28–1.15), which was not seen in the chemotherapy group, thus SCLC-I subtype could be a predictive biomarker rather than prognostic for SCLC immunotherapy. The reasons may be related to the highest immune infiltration and cytolytic activity, the consistently higher expression of 18-gene interferon-γ-related T cell gene expression profile (GEP), and the significantly higher or lower expression level of genes in SCLC-I subtype tumors compared with other subtypes.

**Table 6:** The median overall survival (mOS) and HRs for OS and median OS values between carboplatin/etoposide + atezolizumab (EP + A) and EP + placebo arms in patients from IMpower133 as a collective and by subtype

	EP + A arm		EP + placebo arm		HR (95% CI)
	*N*	mOS (months)	*N*	mOS (months)	
ALL	132	11.6	139	10.1	N/A
SCLC-A	77	10.9	63	10.6	0.807 (0.547–1.189)
SCLC-N	25	10.6	36	9.4	0.631 (0.353–1.129)
SCLC-P	9	9.6	12	6.0	0.595 (0.223–1.589)
SCLC-I	21	18.2	28	10.4	0.572 (0.284–1.15)

*N Number of assessable persons, *N/A* not available

Inflammatory T cell gene expression profile (Tcell_inf_GEP) is a pan-cancer T cell inflammatory gene expression profile consisting of 18 genes. In KEYNOTE-028 (Ott et al. 2019), Tcell_inf_GEP had been demonstrated to be potentially associated with clinical efficacy in immunotherapy for 20 solid tumors, including SCLC. Kanemura et al. (Kanemura et al. 2022) included 135 ES-SCLC patients who received chemotherapy alone (chemo-cohort, *n* = 71) or ICI combination chemotherapy (ICI combo-cohort, *n* = 64) in a retrospective study. And based on tumor PD-L1 expression and CD8^+^ tumor-infiltrating lymphocyte (TIL) density, the above two cohorts were further classified into “inflamed tumors” (PD-L1 CPS ≥ 1% and CD8^+^ TIL density > 85/mm^2^) and all other tumors as “noninflamed tumors”. In the ICI combo-cohort, the mPFS in patients with inflamed tumors (*n* = 7) and noninflamed tumors (*n* = 56) tumors were 10.8 and 5.1 months (*p* = 0.002, HR: 0.26, 95% CI 0.09–0.74), respectively. In contrast, for the chemo-cohort, there were no significant results. Subsequently, further Tcell_inf_GEP analysis of 89 tumor samples (50 cases from the chemo-cohort and 39 cases from ICI combo-cohort) taken demonstrated that inflamed tumors (17 cases) had a higher Tcell_inf_GEP score than noninflamed tumors (72 cases) (*p* < 0.001). Meanwhile, the study also derived frameshift neo-antigen loads from whole-exome sequencing (WES) and found that in the combined ICI cohort, the 12-month PFS rate of tumors with high and low (bounded by the median value) frameshift neo-antigen loads were 16.1% and 0%, respectively. These results suggest that PD-L1 expression, CD8^+^ T cell infiltration, and high frameshift neo-antigen loads are associated with clinical benefit of ICI therapy in ES-SCLC. Besides, it was also verified that Tcell_inf_GEP may play an important role in the more favorable response of inflamed tumors to ICIs. However, in an exploratory biomarker analysis of the phase III KEYNOTE-604 study (Rudin, et al. 2023), Charles M. et al. evaluated Tcell_inf_GEP as a survival associated factor using RNA-seq and found that higher Tcell_inf_GEP was positively correlated with OS in both the placebo group (*p* < 0.005) and the immunotherapy group (*p* = 0.003), with no additional OS benefit from immunotherapy observed. It was suggested that Tcell_inf_GEP may not be a specific biomarker for immunotherapy in SCLC.

Notably, Kanemura et al. (Kanemura et al. 2022) also found that  SRY-related high-mobility-group box 11(SOX11) (*p* < 0.001, false discovery rate (FDR) < 0.001) and myelocytomatosis (MYC) (*p* = 0.02, FDR = 0.06) were the top two up-regulated genes in non-inflammatory tumors relative to inflamed tumors in the above-mentioned study, which suggested that SOX11 and MYC may contribute to poor immune response in SCLC. MYC was further analyzed and patients were divided into high and low MYC cohorts based on median MYC expression for survival analysis. And in the ICI combo-cohort (*n* = 39), longer mPFS (HR: 2.18, 95% CI 1.08–4.40, *p* = 0.028) and higher 12-month PFS rate (4.6% vs. 23.5%) were found in the low-MYC group compared with high MYC. In contrast, there was no significant difference in mPFS (HR: 1.09, 95% CI 0.61–1.94, *p* = 0.77) between the two cohorts in the chemo-cohort (*n* = 50). These results suggest that MYC expression is negatively associated with ICIs efficacy.

To determine whether genomic, transcriptomic, proteomic, and T cell receptor (TCR) sequencing could predict clinical benefits to immune checkpoint blockades (ICBs), Roper et al. (Roper et al. 2021) comprehensively assessed the immunogenomic signatures of tumor samples from 20 patients with relapsed SCLC who received durvalumab combined with a poly (ADP ribose) polymerase (PARP) inhibitor as a discovery cohort. Similar to previous studies, the results suggested that patients had four characteristics that could benefit from ICBs: cytotoxic T cell infiltration, high expression of antigen processing genes, antigen-presenting machinery genes, and low neuroendocrine differentiation. Ultimately, activation of Notch signaling was responsible for low neuroendocrine differentiation and intrinsic tumor immunity in SCLC. The results were confirmed in both clinical validation cohorts and in vitro. Therefore, the findings from this study suggest Notch signaling is the determinant of the SCLC response to ICBs. However, there are still some questions to ponder: First, the study was also based on cohorts of relapsed SCLC patients, either the discovery cohort or the validation cohort, whereas ICBs are now used in the first-line setting in combination with chemotherapy. Second, multiple SCLC molecular subtypes arise from aneuroendocrine cells of origin, and resistance after chemotherapy causes MYC to activate Notch signaling to dedifferentiate neuroendocrine SCLC in a conserved trajectory from ASCL1^+^ to NEUROD1^+^ to the YAP1^+^ nonneuroendocrine subtype (Ireland et al. 2020). Therefore, immunotherapy may be increasingly effective in the evolution trajectory of SCLC.

### Expression of major histocompatibility complex (MHC) -I/II

It was recognized as early as 1985 that the expression of MHC class I molecules (MHC-I) is low in most SCLC cell lines and pathological tissues of patients (Doyle et al. 1985). Zhang et al. (Zhang et al. 2022) analyzed 7 SCLC samples and found overall low expression of MHC-I-related genes in TCs from patients with less immune cell infiltration. Meanwhile, Nguyen et al. (Nguyen et al. 2022) also suggested that epigenetic silencing of MHC-I in SCLC resulted in a poor response to ICBs and found that restoring MHC-I cell surface expression in SCLC could recruit and increase immune cell infiltration, leading to a significant increase in cytotoxic and activated CD8^+^T cells, and enhancing the anti-tumor immune response to ICBs in SCLC. Similarly, Navin et al. (Mahadevan et al. 2021) analyzed a sample of patients with primary SCLC (*n* = 102) using standard chromogenic IHC and multiplexed immunofluorescence and found little or no expression of MHC-I in most cases (72/102). Nearly 15% (15/102) of cases showed high expression of MHC-I, with non-neuroendocrine features. The investigators also collected clinical information on a group of patients (*n* = 31) who had a durable response to ICBs, and found that patients with SCLC with high MHC-I expression had a significantly more durable response to ICBs when stratified by the level of MHC-I expression (*p* = 0.02; HR: 0.13). Thus, MHC-I and the characterization of this non-neuroendocrine state may serve as biomarkers for immunotherapy of SCLC.

Robert et al. (Alspach et al. 2019) found in mice sarcoma model that ICB therapy sensitivity depends on the combination of CD4^+^ and CD8^+^T cells, predicting that even tumor patients with immunogenic MHC-I neo-antigens may not respond to immunotherapy due to the absence of immunogenic MHC-II-restricted CD4^+^T cell antigens. Thus, MHC-II-restricted neo-antigens may have critical functions in antitumor responses and do not overlap with MHC-I-restricted neo-antigens. In a previous study, He et al. (He et al. 2017) confirmed that MHC-II was expressed in NSCLC cell lines and tissues, but no MHC-II expression was found in SCLC. And MHC Class II expression was lower on SCLC TILs than on NSCLC TILs (15.3% vs. 56.7%, *p* < 0.001). At the 2021 World Conference on Lung Cancer (WCLC), Garassino et al. (Garassino et al. 2021) further analyzed exploratory data from the CASPIAN study. A total of 414 patients (52% of the total population) could be evaluated for the human leukocyte antigen (HLA)-I/II genotype (biomarker-evaluable population [BEP]). The incidence of HLA-DQB1*03:01, an MHC class II allele, in BEP was 37%. The MHC class II allele was associated with longer OS in the durvalumab (D) + tremelimumab (T) + EP group (14.9 vs. 10.5 months, HR: 0.59, 95% CI: 0.39–0.88), but not in the D + EP (HR: 0.93, 95% CI 0.63–1.37) or EP (HR: 0.94, 95% CI 0.61–1.40) groups. Therefore, MHC class II molecules may be involved in the inhibition of cytotoxic T lymphocyte antigen 4 (CTLA-4), thereby enhancing the therapeutic efficacy of dual immunotherapy (CTLA-4 + PD-L1) combined with chemotherapy. However, few studies had explored the degree of MHC class II expression on SCLC as well as the correlation between expression and the immune efficacy of SCLC. Therefore, although MHC-I/II molecules are less expressed in SCLC, their expression still has a better predictive effect on the benefit of ICIs, and there are few studies in this field of SCLC, which still needs more research and further exploration.

### Tumor immune microenvironment (TIME) and chemokines

Compared with other tumor types, the TIME of SCLC is affected by low PD-L1 expression, insufficient MHC molecules expression, and dysregulated expression of cluster differentiation antigens, which leads to reduced immunogenicity, decreased TILs, and decreased antigen presentation to TILs, especially CD8^+^ T cells. Meanwhile, cytokines, such as IL-15 secreted by SCLC inhibit CD4^+^T cell proliferation and support regulatory T cells (Tregs) induction. Infiltration of myeloid-derived suppressor cell (MDSC) leads to T cell inactivation and apoptosis, inhibits the activity of natural killer (NK) cells, and induces the differentiation and proliferation of CD4^+^ T cells into Tregs (Tian, et al. 2019; Zhu and Wu 2020). Therefore, the TIME of SCLC has the characteristics of immunosuppression.

In the phase II study of olaparib combined with durvalumab in the treatment of relapsed SCLC, Anish Tho et al. (Thomas, et al. 2019) collected pretreatment and on-treatment (2–4 weeks after treatment) tumor tissue biopsy specimens from the same location to evaluate the dynamic changes of T cell infiltration and PD-L1 expression. Nine of 14 (64%) tumors exhibited an excluded phenotype (CD8^+^T cells in the stroma immediately adjacent/within the tumor). 21% and 14% of tumors exhibited inflamed (CD8^+^T cells in direct contact with the tumor) and desert phenotypes (CD8^+^T cell prevalence low), respectively. The ORR was only 10.5%, and the study did not meet the preset bar for efficacy (35%). However, exploratory biomarker analysis found that tumor responses (2 cases of confirmed responses and one with a systemic response and brain-only progressive disease) were observed in all instances when pretreatment tumors showed an inflamed phenotype. None of the non-inflamed tumors responded to treatment. This study further demonstrates that the immune phenotypes may predict the response of SCLC patients to ICIs. However, the small amount of tissue included in this study was only 19 cases, and this study was a single-arm trial lacking control. Therefore, whether immune phenotypes could be a predictor of immunotherapy in SCLC needs to be confirmed in larger cohorts.

As mentioned above, the SCLC-I subtype and SCLC-P subtype were confirmed to respond better to immunotherapy. Further analysis of the immune microenvironment of the above subtypes showed that compared with other subtypes, not only the abundance of dendritic cells, macrophages, induced-regulatory T cells (iTreg) and CD8^+^ T cells increased significantly, but also the expression of most chemokines, such as CXCL10, CCL17, and CCL18, were up-regulated (Chen et al. 2021).

Chemokines in the body are involved in the development of a variety of cancers, such as by promoting the proliferation and metastasis of cancer cells, regulating the immune microenvironment, etc. These factors play a key determinant in tumor progression, so they have a great influence on the therapeutic efficacy and prognosis of the patient (Vautrot et al. 2021). Tang et al. (Tang et al. 2022) found that C–C Motif Chemokine Ligand 5 (CCL5) expression status correlated with survival prognosis (HR: 0.62, 95% CI 0.39–0.96, *p* = 0.04) in SCLC patients treated with immunotherapy. And further studies have found that CCL5 expression significantly affects the TIME. CD8^+^T cells, memory B cells, dendritic cells, M1 macrophages, and NK cells were positively correlated with CCL5 expression, while M2 macrophages were negatively correlated with CCL5 expression. There is also a positive correlation between the expression of CCL5 and PD-L1 (*p* < 0.05). In addition, the cohort analysis found that patients with high CCL5 expression often have other high levels of common immune checkpoints, such as PD-L1, CTLA-4, T cell immunoglobulin mucin 3 (TIM-3), lymphocyte-activating gene 3 (LAG3), etc. Taken together, it is indicated that a wide range of interactions exist between CCL5 and patients with high CCL5 expression who are predicted to respond better to immunotherapy. Research in future is required since these findings are the product of data mining and validation processes based on very limited samples.

### Circulating tumor DNA (ctDNA)

CtDNA is composed of DNA fragments released by the TCs into the circulation of the blood cell system, and its relative abundance was quantified by the variant allele fraction (VAF) of the most represented mutation (Rudin et al. 2021; Nong et al. 2018). Herbreteau et al. (Herbreteau, et al. 2020) enrolled 46 SCLC patients treated with second-line atezolizumab and 22 with conventional chemotherapy and found that 49/68 patients (70.6%) had detectable baseline ctDNA, indicating that the ctDNA detection rate is still high even before the second line in SCLC. All patients with detection of ctDNA mutations had a significantly lower disease control rate (DCR) at week 6 than patients without ctDNA mutation (29.5% versus 58.8%, respectively, *p* = 0.030), regardless of the treatment modality. Analyzed separately according to treatment subgroups, after 6 cycles of treatment, the detection of ctDNA mutations was not associated with any difference in DCR in patients treated with chemotherapy (64.3% versus 71.4%; *p* = 0.672), while patients treated with immunotherapy had a significantly lower DCR when the ctDNA mutations was detected (13.3% vs. 50%; *p* = 0.0145). The result suggested ctDNA mutation status after immunotherapy could be predictive biomarker for efficacy of SCLC patients who received second-line immunotherapy.

As ctDNA has better timeliness and accessibility than tissue specimens, it is helpful to understand the dynamic changes of ctDNA, and to further explore the relationship between the characteristics of dynamic changes of ctDNA during the course of treatment and the efficacy of treatment and survival outcomes. Sivapalan et al. (Sivapalan et al. 2023; Pellini and Chaudhuri 2023) studied the correlation between ctDNA dynamics and survival outcome in patients with ES-SCLC. In order to improve the sensitivity of detecting ctDNA molecular reaction, tumor-derived sequence alterations and plasma aneuploidy were evaluated serially and combined to assess changes in total cell-free tumor load (cfTL). Plasma samples from 33 patients (17 cases receiving chemotherapy and 16 case receiving chemotherapy combined with immunotherapy) were analyzed longitudinally at least three time points (before treatment, during treatment, and at clinical progression). ctDNA dynamics were then divided into three groups based on the results: molecular response (persistent complete elimination of cfTL), relapse after molecular response followed by recrudescence (initial elimination of cfTL, elevation at final time points), and molecular progression (persistence of cfTL at all time points). Patients with molecular response assessed against longitudinal dynamic changes in cfTL had longer OS and PFS than the other two types (mOS: OS not reached vs. 12.35 vs. 6.48 months, respectively, *p* = 0.0006 and mPFS: PFS not reached vs. 6.18 vs. 1.74 months, respectively, *p* < 0.0001). Multifactor regression analysis with other factors further demonstrated that ctDNA molecular responses was still an important predictor of OS (molecular response vs. molecular progression HR: 0.09, 95% CI 0.02–0.42, *p* = 0.002; molecular response f/b recrudescence vs. molecular progression HR: 0.14, 95% CI 0.04–0.48, *p* = 0.002) and PFS (molecular response vs. molecular progression HR: 0.02, 95% CI 0.00–0.16, *p* < 0.001; molecular response f/b recrudescence vs. molecular progression HR = 0.05, 95% CI 0.01–0.26, *p* < 0.001).And compared with imaging evaluation, ctDNA can better predict OS and PFS of SCLC patients (36 m OS: AUC, 0.80 vs 0.72; 12 m PFS: AUC, 0.84 vs 0.79).

With the development of liquid biopsy technology, the predictive potential of ctDNA in other aspects is also worth further exploration (Heitzer et al. 2019). Nie et al. (Nie et al. 2022) demonstrated that ctDNA in blood has a potential impact on the predictive efficacy of bTMB, so they defined ctDNA-adjusted bTMB and tested it in a cohort of advanced NSCLC patients (*n* = 853) who received atezolizumab or docetaxel after failure of platinum-based therapy. They found the ctDNA-adjusted bTMB showed better predictive performance than unadjusted bTMB (AUC: 0.63 vs 0.46, *p* = 0.013). In contrast to the chemotherapy group, high ctDNA-adjusted bTMB was significantly associated with improved durable clinical benefit (DCB) (*p* < 0.001) and ORR (*p* = 0.020), and the interaction *P* values for atezolizumab vs. docetaxel treatment were positive for OS (*p* = 0.016) and PFS (*p* = 0.002), which indicated that high ctDNA-adjusted bTMB might predict better outcomes with ICIs treatment. Thus, the combination of ctDNA with other biomarkers may show better predictive value. Moreover, ctDNA can be used to comprehensively characterize the tumor genome to identify gene mutations associated with immunotherapy sensitivity. Guibert et al. (Guibert et al. 2019) collected plasma specimens from NSCLC patients before receiving ICIs treatment and further graphed the molecular profile of ctDNA. They found that patients harboring STK11 or PTEN mutations derive poor benefit from PD-1 inhibitors compared with patients who don’t (HR: 4.7, *p* = 0.003 for STK11; HR: 8.9, *p* = 0.09 for PTEN). And patients with a KRAS or TP53 transversion mutation showed better responses compared to patients without (HR: 0.36, *p* = 0.011 for TP53 Tv; HR: 0.46, *p* = 0.11 for KRAS Tv).

In conclusion, ctDNA has the potential to predict the efficacy and prognosis of tumor immunotherapy, but there is still insufficient evidence for its application in patients with SCLC. Previous studies mostly focused on prognostic data and lacked predictive data, so sufficient clinical studies are still needed for further exploration.

## Conclusion

In summary, the exploration of biomarkers for immunotherapy of SCLC is still very difficult, and it is clear that conventional predictive biomarkers are not suitable for SCLC. At present, the molecular subtypes defined from transcription factors may have some guiding significance, which still needs to be confirmed by prospective clinical studies. In addition, the ctDNA positivity rate of SCLC is higher than that of other tumor types, which can also solve the dilemma of the difficulty of obtaining specimens of SCLC tissues. And the dynamic change of ctDNA also has great potential to predict the curative effect of SCLC, which is worth further clinical exploration.

## Data Availability

Data available on request from the authors.
